# Severe Hyponatremia and Syndrome of Inappropriate Antidiuretic Hormone Secretion After Kambô Ritual

**DOI:** 10.1002/ccr3.71069

**Published:** 2025-10-04

**Authors:** Thomas Kelly, Angela Saponaro, James Longenbach, Tomer Finkelberg, Christine Giesa, Eric Kleiman, Hyra Syed, Pierre Frederique, Richard J. Hamilton

**Affiliations:** ^1^ Department of Medicine Crozer Chester Medical Center Upland Pennsylvania USA; ^2^ Department of Emergency Medicine Crozer Chester Medical Center Upland Pennsylvania USA; ^3^ Department of Emergency Medicine Drexel University College of Medicine Philadelphia Pennsylvania USA; ^4^ Philadelphia College of Osteopathic Medicine Philadelphia Pennsylvania USA

**Keywords:** electrolyte disturbances, hyponatremia, Kambô poisoning, peptide toxicity, SIADH

## Abstract

Kambô poisoning can cause severe hyponatremia, neurologic effects, and gastrointestinal symptoms. Clinicians need to determine whether the derangements are the result of ritualistic consumption of water or toxin‐induced syndrome of inappropriate antidiuretic hormone secretion (SIADH). An appropriate workup for SIADH guides electrolyte correction and leads to resolution of neurologic and systemic effects.

## 1 Case History

**FIGURE 1:**
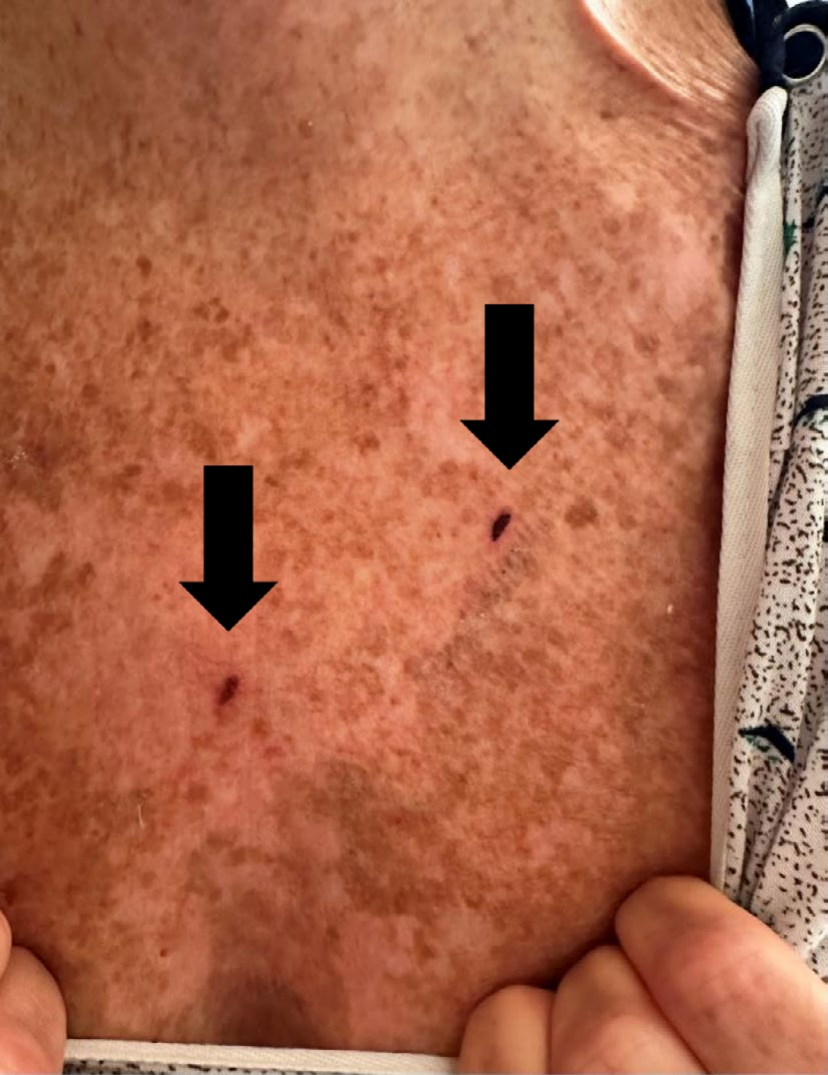
Chest burn wounds where poison was applied.

**FIGURE 2:**
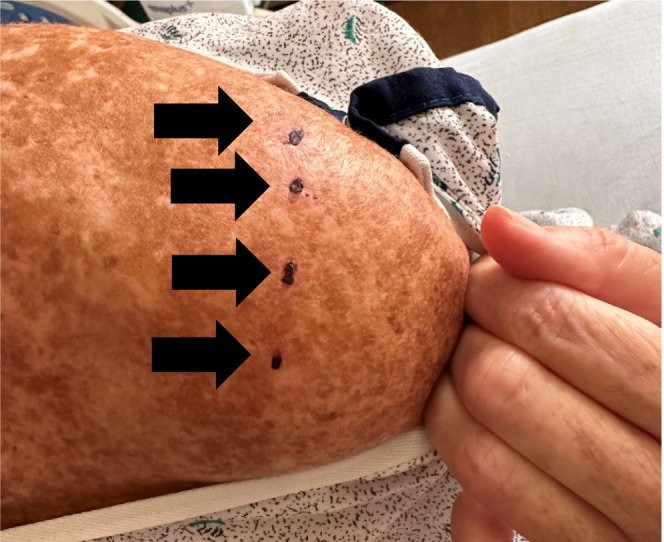
Shoulder burn wounds where poison was applied.

Emergency medical services (EMS) transported a 64‐year‐old female with a history of alcohol use disorder to the emergency department due to acute mental status changes after a Kambô ritual. She had no other past medical history and was taking no medications. A self‐described shaman arrived with the patient. She had ingested a gallon of water, and the shaman created small burns on her chest and shoulder (Figures 1 and 2) to which he applied frog venom. Soon after the venom application, the patient experienced expected symptoms of emesis and diarrhea. Six hours later, she developed muscle cramps and consumed a banana to treat presumed hypokalemia. Shortly after eating the banana, the patient became unresponsive, prompting the shaman to call EMS.

Upon arrival at the emergency department, the patient displayed intermittent agitation and unresponsiveness while still protecting her airway. Reflexes were normal without clonus. Initial laboratory results showed leukocytosis (14.7 k/mm^3^), hyponatremia (118 mmol/L), normal potassium (4.3 mmol/L), and mild lactic acidosis (2.9 mmol/L). Medical staff administered 4 mg intramuscular midazolam to manage her agitation and facilitate imaging and intravenous access. A urine drug screen, obtained 4 h after the administration of midazolam, indicated the presence of benzodiazepines. A serum analysis later confirmed the presence of only midazolam and its metabolites with no other drugs of abuse. Acetaminophen, ethanol, and salicylate levels were below the level of detection. Radiology reported the computed tomography (CT) scan of the head as normal and the chest radiograph as vascular congestion or bronchitis.

## 2 Differential Diagnosis, Investigations and Treatment

Given the patient's presentation after a Kambô ritual and the associated symptoms, the key differential diagnoses considered were Kambô ritual (frog venom) toxicity, electrolyte imbalance, alcohol withdrawal, serotonin syndrome, sepsis, and metabolic derangements leading to lactic acidosis.

Kambô is a traditional ritual that involves the application of frog secretion (from the *Phyllomedusa bicolor* ) to small burns on the skin. The venom contains potent bioactive peptides that affect the cardiovascular and nervous systems, leading to symptoms such as vomiting, diarrhea, agitation, muscle cramps, and unresponsiveness [1, 2]. The patient's ingestion of water followed by the ritual and subsequent symptoms (vomiting, diarrhea, muscle cramps, agitation) is consistent with Kambô toxicity. The subsequent unresponsiveness may represent a severe systemic response to the venom or its effects on electrolyte balance. Hyponatremia (118 mmol/L) is a key finding, and while the potassium level is normal, the patient's electrolyte disturbances could be contributing to her symptoms. Severe hyponatremia can lead to altered mental status, agitation, and confusion. Given her history of alcohol use disorder, alcohol withdrawal is a possible contributing factor to her altered mental status. The timing of her mental status change, six hours after the ritual, could correspond with the onset of withdrawal, especially if she has not been drinking regularly. Symptoms such as agitation, confusion, and hallucinations can occur during withdrawal.

Although less likely, serotonin syndrome could be considered if the patient had used any psychoactive substances during the Kambô ritual, especially if they were combined with other drugs (e.g., alcohol or stimulants). Symptoms typically include agitation, confusion, hyperreflexia, and autonomic dysregulation, which could explain her agitation and agitation‐related symptoms. Additionally, if other drugs (e.g., recreational substances) were involved in the ritual or were unknowingly ingested, these substances might explain her altered mental status and agitation. The patient's leukocytosis (14.7 k/mm^3^) raises the concern for infection, though no obvious source of infection has been identified. The stress of the ritual, combined with the potential for compromised skin integrity from the burns, could predispose her to infections or systemic inflammatory response syndrome (SIRS). Finally, the mild acidosis with elevated lactate (2.9 mmol/L) seen on initial laboratory tests could indicate tissue hypoperfusion or hypoxia, endogenous catecholamine release, potentially from either a toxic reaction to the frog venom, alcohol withdrawal, or sepsis. The patient may have experienced a reduction in effective circulation due to electrolyte abnormalities, affecting organ perfusion and leading to lactic acid accumulation.

Urinalysis returned with a urine specific gravity of 1.104, spot urine osmolality of 574 mosm/kg, and urine sodium of 157 mmol/L—all of which confirmed the presence of SIADH. She was admitted to the ICU and received a total of 891 mL of 3% hypertonic saline over 24 h, which raised her sodium from 119 to 129 mmol/L. Thyroid studies were normal and adrenal crisis/glucocorticoid deficiency was considered less likely given the response to treatment. Her mental status improved after 24 h, but she continued to experience headache, dizziness, loss of balance, and short‐term memory loss. These symptoms progressively resolved before her discharge on day five with sodium at 135 mmol/L and no residual deficits. However, she remained amnestic to the events that occurred between the ritual and her waking up in the hospital.

## 3 Conclusion and Results

One month follow‐up revealed no significant sequelae. The patient consented to the publication of this case report.

## 4 Discussion

Kambô poisoning presents a unique challenge to emergency medicine [1, 2]. The venom contains a complex mixture of hundreds of biologically active peptides, including dermorphins (opioid receptor agonist), deltorphins (opioid receptor agonist), dermaseptins (antimicrobial peptides), phyllomedusin (tachykinin), phyllokinin (bradykinin), and sauvagine (vasodilator). Each of which exerts distinct physiological effects. The venom is derived from the secretions of *Phyllomedusa bicolor* , an Amazonian tree frog. These peptides are likely responsible for the psychoactive effects, but the reported acute and subacute effects of Kambô more so resemble stimulants rather than those of substances with mu‐opioid activity. This suggests that other compounds, or complex interactions between vegetative, neuro‐endocrinological, and psychological effects of peptides may be responsible for the biological correlates of Kambô [3, 4, 5]. Gastrointestinal distress, including vomiting and diarrhea, is common in Kambô exposure and is often followed by more severe symptoms such as seizures, coma, or electrolyte disturbances [6, 7]. In this case, the patient developed profound hyponatremia, likely secondary to SIADH induced by the venom's complex interaction with the hypothalamic–pituitary axis.

Previous reports have documented Kambô‐associated hyponatremia and SIADH, though the precise mechanism remains unclear. Phyllomedusin, a vasoactive peptide, may play a role in promoting antidiuretic hormone release, contributing to the development of SIADH [6]. Additionally, excessive water consumption during the ritual likely exacerbated the patient's hyponatremia. Water consumption is a component of native rituals, particularly among Amazonian tribes such as the Matsés. This practice of water consumption is generally done to induce vomiting, which is thought to help cleanse the body of toxins. Excessive water consumption is more commonly associated with non‐native practitioners and a modern adaptation of the ritual. Clinicians may mistake the hyponatremia after the Kambô ritual as the result of deliberate overconsumption of water; however, this case highlights the importance of suspecting SIADH.

To date, reports of acute poisonings from Kambô have shown wide‐ranging effects including dermatomyositis, toxic hepatitis, psychosis, hyponatremia, SIADH, myocardial infarct, and even sudden death [8]. Management of Kambô poisoning remains largely supportive, with particular emphasis on electrolyte correction and monitoring for neurologic sequelae. In this case, hypertonic saline therapy was effective in correcting hyponatremia and restoring the patient's neurologic function. Early recognition of SIADH and judicious use of hypertonic saline when marked hyponatremia presents with severe neurologic symptoms is critical in managing severe hyponatremia associated with Kambô exposure.

This case highlights the importance of recognizing the potential toxicities of Kambô, particularly the risk of severe electrolyte imbalances such as hyponatremia and SIADH. Healthcare providers should be aware of the growing prevalence of Kambô use and its associated complications. Prompt identification and treatment of electrolyte disturbances are essential to prevent long‐term neurologic damage from overcorrection of SIADH.

## Author Contributions

**Thomas Kelly:** writing – original draft, writing – review and editing. **Angela Saponaro:** writing – original draft. **Tomer Finkelberg:** writing – original draft. **James Longenbach:** writing – original draft. **Eric Kleiman:** writing – original draft, writing – review and editing. **Hyra Syed:** writing – original draft, writing – review and editing. **Christine Giesa:** writing – original draft, writing – review and editing. **Pierre Frederique:** writing – review and editing. **Richard J. Hamilton:** conceptualization, project administration, writing – original draft, writing – review and editing.

## Ethics Statement

This study was conducted in accordance with the Declaration of Helsinki and was approved by the Crozer Chester Medical Center Institutional Review Board.

## Consent

Written informed consent was obtained from the patient for publication.

## Conflicts of Interest

The authors declare no conflicts of interest.

## Data Availability

The data that support the findings of this study are available on request from the corresponding author. The data are not publicly available due to privacy or ethical restrictions.
